# Effects of COVID-19-Associated Infection Control on the Pattern of Infections Imported by German Soldiers and Police Officers Returning from Predominantly Tropical Deployment Sites

**DOI:** 10.3390/idr15060070

**Published:** 2023-12-11

**Authors:** Dorothea Franziska Wiemer, Matthias Halfter, Ulrich Müseler, Marius Schawaller, Hagen Frickmann

**Affiliations:** 1 Tropical Medicine and Infectious Disease Unit, Medical Department, Bundeswehr Hospital Hamburg, 20359 Hamburg, Germany; wiemer@bnitm.de (D.F.W.); matzehalfter@gmx.de (M.H.); 2 Medical Service, German Federal Police, 14473 Potsdam, Germany; ulrich.mueseler@polizei.bund.de; 3 Medical Department, Helios Clinics Uelzen, 29525 Uelzen, Germany; marius@schawaller-online.de; 4 Department of Microbiology and Hospital Hygiene, Bundeswehr Hospital Hamburg, 20359 Hamburg, Germany; 5 Institute for Medical Microbiology, Virology and Hygiene, University Medicine Rostock, 18057 Rostock, Germany

**Keywords:** COVID-19, SARS-CoV-2, pandemic, tropics, deployed soldiers, deployed police officers, surveillance, infectious disease screening

## Abstract

In response to the COVID-19 pandemic, German public health authorities launched various infection control procedures. In line with this, anti-pandemic infection control was also implemented for German military and police deployments. The presented study assessed the impact of this increased infection control effort on deployment-associated infections in a holistic approach. To do so, the results of post-deployment assessments offered to German soldiers and police officers at the Department of Tropical Medicine and Infectious Diseases of the Bundeswehr Hospital Hamburg obtained during the pandemic period were compared to the results recorded during the pre-pandemic period in an exploratory, hypothesis-forming comparative study. In total, data from 1010 military deployments and 134 police deployments, predominantly to the African or the Eastern Mediterranean WHO regions, were included in the analyses. In the main results, a significant decrease in gastroenteritis in deployed soldiers (20.1% versus 61.3%, *p* < 0.0001) and at least a trend in the same direction in deployed police officers (25.7% versus 35.4%, *p* = 0.4026) were shown for the pandemic period, while no consistent tendency into the one or the other direction was detectable for febrile illness on deployment. In contrast to the finding of less frequently reported deployment-associated gastroenteritis, the detection rates of enteric microorganisms after deployment, including poor hygiene-related colonization with apathogenic protozoa, remained unchanged. Regarding non-enteric infections, the numbers of serologically confirmed malaria cases on deployment and as expected, due to increased airway protection, *Mycobacterium tuberculosis*-specific immune-conversion dropped significantly with *p* = 0.0037 and *p* = 0.009, respectively. As a side finding, soldiers and police officers with post-deployment medical assessments were more likely to be older and male during the pandemic compared to the pre-pandemic period. In summary, only minor changes in deployment-associated infection and colonization rates were seen in response to the increased infection control procedures during the pandemic period, apart from respiratory infections. In particular, the clinical finding of less gastroenteritis on deployment was not matched by a concordant decline in poor hygiene-related enteric colonization with apathogenic protozoa in the soldiers’ guts, indicating that the fecal–oral transmission risk remained basically the same.

## 1. Introduction

During the COVID(Corona Virus Disease-)-19 pandemic, strict infection control measures were globally enforced in an attempt to keep the estimated numbers of pandemic-associated excess deaths, which were observed even in resource-rich industrialized countries [1], as low as possible. Military facilities were not exempt from this rule. Recently, several military medical researchers provided reports on how COVID-19 infection control was implemented at military units on deployment, in the home country as well as on armed Navy vessels [2,3,4,5]. As reported for pandemic management on a NATO (North Atlantic Treaty Organization) airbase in the Afghan deployment setting, the standard procedures comprised the isolation of infected individuals, quarantine for contacts, mask-based airway protection, and so-called “social distancing”, symptom screening, active contract tracing, as well as extensive (or, as called by the authors, “aggressive”) and obligatory molecular diagnostic on-site screening to identify asymptomatic or hypothetically dissimulating virus carries [6]. As reported by Israeli military medical researchers, quarantine orders resulting from contact tracing especially had a considerable impact on the operational readiness of their forces. Balanced against the relatively mild medical impact of SARS-CoV-2 on their predominantly young and healthy soldiers, they advocated for more liberal strategies in similar situations [7]. US (United States-) American military medical researchers calculated differences in quarantine costs, with quarantining based on geographic movement being more costly than quarantining based on close contact with infected individuals. The lost time on active duty per contained infection was nevertheless considerable [8].

Irrespective of any normative interpretations on the appropriateness of chosen anti-pandemic measures, the experience reported by the Israeli and US American colleagues above [7,8] shows that strict infection control measures necessarily come with a price defined by undesired effects apart from infectious disease transmission prevention. Partly controversially discussed undesired effects among military personnel comprised overweight and diabetic diathesis due to reduced physical activity [9,10,11], age-dependent and predisposition-dependent increases in suicide rates [12,13,14], compensatory increases in risky sexual activity [15,16], affective disorders [17], higher rates of gambling problems [18] and considerable burn-out rates in military medical health staff [19]. Typical for such multi-factorially influenced medical conditions, it is difficult to define which effects have been in response to the medical impact of COVID-19 itself or to side effects of the anti-pandemic response on the individuals’ social life and well-being.

Indirect support of the latter option is provided by assessments of the beliefs and attitudes of military personnel towards the COVID-19 pandemic, which showed that the spectrum of attitudes was considerable and, by far, not all soldiers considered the disease-associated threat as serious [20,21]. It is likely that negative attitudes towards restrictive infection control measures might have been facilitated by the fact that soldiers were much less endangered than civilians by COVID-19 in terms of both the need for intensive care treatment and death [22], which is a typical “healthy worker effect”. Preventive strategies, including COVID-19 testing of military personnel with partly insufficiently reliable assays as described elsewhere [23] may also have negatively influenced the soldiers’ trust in enforced infection control strategies. Insofar, it is not surprising that in spite of the fact that soldiers are in general both particularly affected by various infectious disease transmission risks [24] and particularly predisposed to participating in infectious disease spread [25], US American military medical researchers recently identified poor adherence to infection control procedures as a major risk of infectious disease spread among and by military personnel [26].

Apart from soldiers, police officers were shown to be at a particularly high risk of acquiring SARS-CoV-2 infections in studies from Europe, South America and the Indian subcontinent [27,28,29,30]. High occupational exposure risks were considered as a likely explanation [28]. Nevertheless, investigators from Poland were able to demonstrate that consequent adherence to infection control procedures like prolongated hand washing, wearing face masks and physical distancing reduced SARS-CoV-2 (Severe Acute Respiratory Syndrome Corona Virus 2) infection rates in police officers as well [30].

Systematic assessments on the effects of the pandemic-associated infection control procedures of both deployed military personnel and deployed police officers are, unfortunately, scarce. This also applies to effects on infectious diseases apart from COVID-19. From civilian assessments, it is known that other infectious diseases were also considerably less frequently recorded due to the pandemic-associated enforcement of stricter infection control procedures, a phenomenon which was even believed to affect immunity at the subpopulation level [31].

In the study presented here, we aimed to investigate hypothetical differences in the results of post-deployment assessments for German soldiers and police officers returning from predominantly tropical deployments regarding diagnosed infectious diseases in the pre-pandemic period and in the pandemic period. Applying this holistic approach, the hypothesis was tested whether or not increased infection control procedures during the pandemic period have led to an overall reduced infectious disease load in the deployed forces.

## 2. Materials and Methods

### 2.1. Study Design and Definitions

The assessment was designed as an exploratory, hypothesis-forming comparative study, comparing anonymized post-deployment assessment results of German soldiers and police officers returning from predominantly tropical deployments during the pre-COVID-19 pandemic period (referred to as “pre-pandemic period” in the following) and during the COVID-19 pandemic period (referred to as “pandemic period” in the following). In minor deviation from the WHO definition, which declared the end of the COVID-19 pandemic not earlier than at the beginning of May 2023, only the 2020–2022 period was defined as the “pandemic period” for this study. The reason was that strict anti-pandemic infection control procedures partly deeply interfering with individual freedom of movement were considerably lowered by German public health authorities starting already at the beginning of the year 2023 [32]. Accordingly, only the 2020 to 2022 period, during which the strictest population-based infection control procedures applied [32], was considered the “pandemic period” in terms of the post-deployment assessment of German soldiers and police officers. Even during this “pandemic period”, infection control procedures were frequently adapted and not always the same [32], both in Germany and at the deployment sites, where regulations as valid in Germany were applied as consequently as regionally feasible. Such time- and region-specific differences were not specifically addressed in the assessment presented here. Instead, the whole pandemic period ranging from 2020 to 2022 was holistically considered as a period of generally increased infection control efforts on deployment without further differentiation. This simplification was necessary in order to provide meaningful case numbers, at least for a holistic assessment, thus accepting associated limitations as detailed in the discussion.

For organizational reasons, the post-deployment screening procedures for deployed German soldiers and police officers were slightly different as detailed in the following.

### 2.2. Post-Deployment Assessment Offered to Returned Soldiers

Post-deployment outpatient assessments after military tropical deployments were offered starting in 2006 at the Tropical Medicine & Infectious Disease Department of the Bundeswehr Hospital Hamburg, located at the Bernhard Nocht Institute for Tropical Medicine Hamburg, which is the German National Reference Centre for Tropical Pathogens. Infrequently, German soldiers returning from non-tropical deployments asked for appointments as well. Case files of German soldiers with post-deployment assessments between 2006 and 2022 were anonymously analyzed in a way comparable to a previous cross-sectional study [33]. Only a minority of military returnees presented with medical disorders related to their deployment, while the majority presented just for the exclusion of such medical conditions. Recorded patient-specific data included age, sex and site of deployment. Routinely documented elements of medical history comprised fever or diarrhea on deployment and, if applicable, the drugs used for antimalarial chemoprophylaxis. For infections with increased likeliness of initially asymptomatic or mild clinical courses, diagnostic assessments were routinely offered. In other cases, symptom-triggered specific diagnostic approaches were added based on clinical suspicion by the physician in charge only. Exposition at a deployment site in an area of endemicity was likely a general diagnostic prerequisite. Serological screenings comprised assessments of antibodies against filariae, *Schistosoma* spp. and *Strongyloides stercoralis* as provided by the Bernhard Nocht Institute for Tropical Medicine Hamburg. Real-time PCR targeting diarrheagenic bacteria, protozoa and helminths was first offered based on diagnostic in-house assays and was switched to commercial assays with comparable diagnostic accuracy within the pandemic period in compliance with the demands of the European in vitro diagnostics regulation (IVDR) as described elsewhere [34]. Microscopy in stool for enteric protozoa and helminth eggs was added at the German National Reference Centre for Tropical Pathogens in Hamburg, Germany. Symptom-triggered diagnostic assessments during the study period comprised, e.g., serology targeting antibodies against *Leishmania* spp. (genus-specific), *Plasmodium* spp. and *Yersinia enterocolitica*; next to interferon-gamma release assays (IGRA) to record immunologically relevant contact to *Mycobacterium tuberculosis* complex; culture-based growth of diarrheagenic bacteria like *Campylobacter* spp. and salmonellae as well as microscopy for *Plasmodium* spp. in blood and *Leishmania* spp. (genus-specific) in bioptic material. While serology is unsuited for the diagnosis of acute malaria, immunofluorescence targeting plasmodium-specific antibodies was conducted for the confirmation or exclusion of reported likely malaria cases that had been diagnosed and treated on deployment. Laboratory diagnosis was not only offered for soldiers after deployment who presented for returnee assessments usually 8–12 weeks after deployment but also for deployed soldiers for whom their physician had just sent diagnostic samples.

### 2.3. Post-Deployment Assessment Offered for Returned Police Officers

Since 2015, first starting as a study approach and then included in an inter-ministerial administrative assistance project, molecular diagnostic assessment of stool samples was offered to German police officers deployed to tropical United Nations (UN) missions on a strictly voluntary basis prior as well as 8–12 weeks after deployment as described in a previous cross-section study [35]. Comparable clinical data as recorded for the deployed soldiers, i.e., age and sex of the patient, deployment site, as well as gastrointestinal and febrile symptoms, were documented on the provided sample sheets for the laboratory. The stool samples of the police officers were subjected to the same molecular diagnostic assessment as mentioned above for the soldiers [34]. Other diagnostic approaches were not applied.

### 2.4. Inclusion and Exclusion Criteria

All available datasets within the study period were included in the assessment. Incompleteness of datasets was no exclusion criterion.

### 2.5. Statistics

Considering low overall case numbers, the obtained results were only descriptively compared, and the assessment was restricted to simple statistical approaches like the calculation of the odds ratios. Significance was calculated with Fisher’s two-sided exact test and odds ratio-associated 95% confidence intervals (95%-CI) using the approximation of Woolf. The calculations were performed by applying the software GraphPad InStat, version 3.06, 32-bit for Windows (GraphPad Software Inc., San Diego, CA, USA).

### 2.6. Ethical Clearance

Ethical clearance for anonymized retrospective assessment without informed consent was provided by the Ethics Committee of the Medical Association of Hamburg, Germany, for the deployment returnees (reference number WF-019/17) in line with German national laws. The study was conducted in accordance with the Declaration of Helsinki and all its amendments.

## 3. Results

### 3.1. Study Populations and Deployment Sites in the Pre-Pandemic and the Pandemic Periods

During the whole study period from 2006 to 2022, a total of 924 soldiers returning from 1.010 deployments, thus resulting in 1.010 included returnee examinations, was assessed. Within the assessment period from 2015 to 2022, 134 returnee assessments could be included from a total of 171 police deployment events, for which pre- and post-deployment screenings were offered.

During the pandemic period, both the numbers per year of medically assessed military or police deployment events in total and the numbers per year of medically assessed military or police returnees dropped slightly to moderately, as indicated in Table 1 and Table 2. During the pandemic period, assessed military or police returnees tended to be older and more likely of male sex compared to the pre-pandemic period (Table 1 and Table 2).

**Figure A1:**
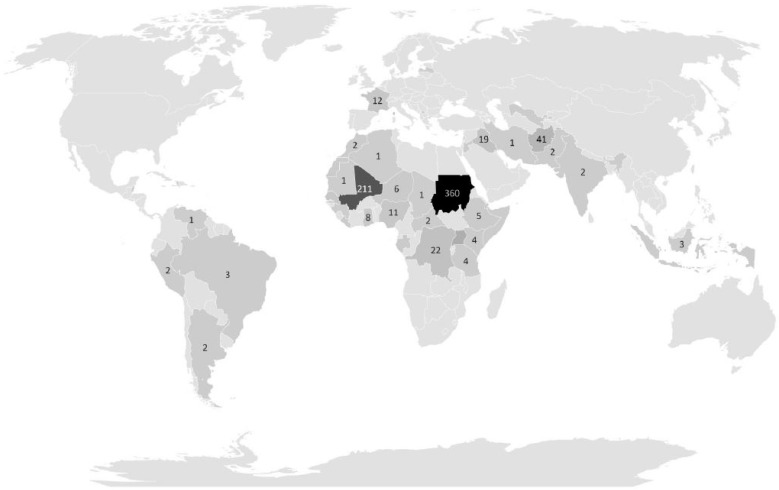
Reported military deployment sites in the pre-pandemic period (2006–2019). Darker grey shades indicate higher proportions of assessed deployed soldiers.

**Figure A2:**
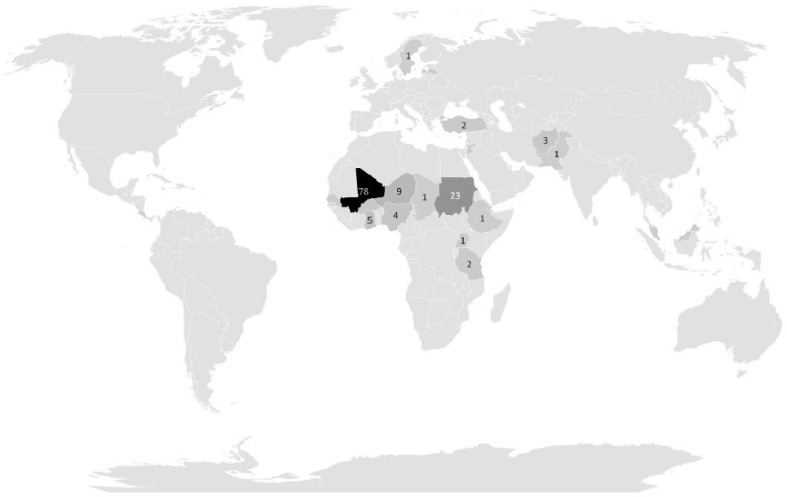
Reported military deployment sites in the pandemic period (2020–2022). Darker grey shades indicate higher proportions of assessed deployed soldiers.

**Figure A3:**
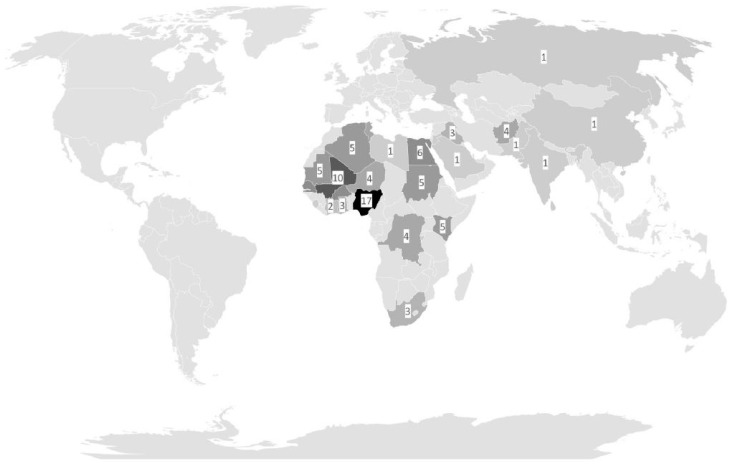
Reported police deployment sites in the pre-pandemic period (2015–2019). Darker grey shades indicate higher proportions of assessed deployed police officers.

**Figure A4:**
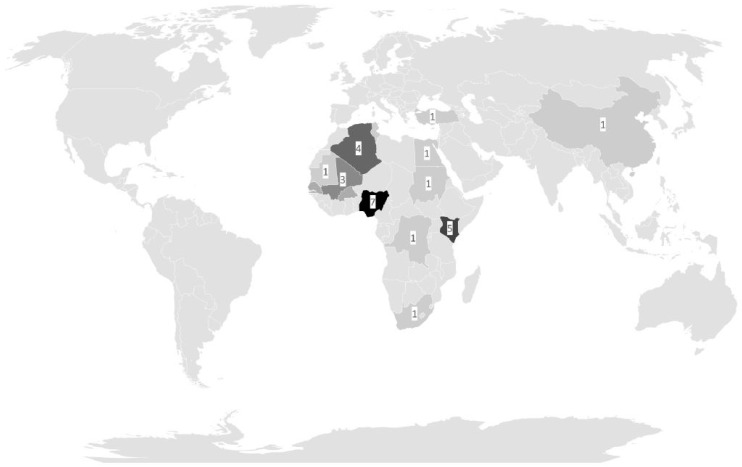
Reported police deployment sites in the pre-pandemic period (2020–2022). Darker grey shades indicate higher proportions of assessed deployed police officers.

As shown in Table 3, military and police returnees from a broad variety of deployment sites but with a proportional dominance of Sub-Saharan African deployment settings were assessed with minor differences in the pre-pandemic and the pandemic period. In both periods, intermediate or high proportions of assessed military returnees had been deployed in Mali or Sudan (including South Sudan), while an intermediate proportion of soldiers returning from Uganda had only been observed in the pre-pandemic period. Focusing on the medically assessed police deployments, intermediate or high proportions comprised Burkina Faso, Mali, Nigeria and Senegal in both periods, while intermediate proportions of deployment assessments were recorded for Egypt solely in the pre-pandemic period as well as for Algeria and Kenya solely in the pandemic period. In addition to the details provided in Table 3, graphic visualizations of the dislocation of the medically assessed returnees from military and police deployments during the pre-pandemic and pandemic period are provided in Appendix A, Figure A1, Figure A2, Figure A3 and Figure A4.

### 3.2. Proportions of Reported Fever and Gastroenteritis on Deployment

The odds ratios for fever on deployment in comparison with the pre-pandemic and the pandemic periods pointed in different directions for German soldiers and police officers without significance. In contrast, there was a significance for less gastroenteritis in deployed soldiers during the pandemic period and a non-significant trend for deployed police officers in the same direction. Details are provided in Table 4 and Table 5.

### 3.3. Direct or Indirect Proof of Infections or Microbial Colonization

Regarding the direct proof of pathogens in German soldiers and police officers after deployment, no significant differences between the pre-pandemic and the pandemic periods were recorded. Regarding indirect proof of deployment-associated infections, which was offered for deployed soldiers only, there was significance for less antibody-based proof of previous plasmodial infections and less interferon-gamma-release-assay-based confirmation of immunologically relevant contact to *Mycobacterium tuberculosis* complex or antigenically closely related non-tuberculous mycobacteria. Details are provided in Table 6 and Table 7. As indicated in Appendix A and Table A1 and Table A2, most deployment-associated infections or microbial colonization events were imported by both soldiers and police officers either from the African or from the Eastern Mediterranean WHO (World Health Organization) region. As shown in Appendix A, Table A3 and Table A4, associations of microbial detections with fever and gastroenteritis were more likely to reflect the overall occurrence of these symptoms in the study populations rather than to suggest clear-cut etiological associations.

### 3.4. Antimalarial Prophylaxis on Military Deployments in the Pre-Pandemic and Pandemic Period

Monitoring of antimalarial chemoprophylaxis in the course of the post-deployment assessments was offered to soldiers only. The comparison of the pre-pandemic and the pandemic periods indicated a relative decline in mefloquine use and an associated relative increase in atovaquone-proguanil-based chemoprophylaxis. Details are provided in Table 8.

## 4. Discussion

The study was conducted to assess the potential impact of pandemic management strategies as enforced in Germany between 2020 and 2022 in response to the COVID-19 pandemic on the results of post-deployment screening approaches for German soldiers and police officers returning from predominantly tropical deployments. The hypothesis was that the increased infection control focus during the pandemic period might have affected the occurrence of non-respiratory infections as well. Civilian assessments had pointed in this direction before [36]. The assessment led to a number of results.

First, there was a minor to moderate decline in the number of post-deployment medical assessments both for German military and police officers during the pandemic period. Within this period, medically assessed personnel were older and more likely to be male than female. During the whole study period, assessed returnees had predominantly been deployed to Sub-Saharan African deployment sites prior to their post-deployment assessments. It remains unclear how far pandemic-associated conditions affected the soldiers’ or police officers’ readiness to participate in the voluntary post-deployment assessments, potentially being associated with fewer consultations of returnees with personal perception of low infection risks on deployment.

Second, the calculated odds ratios did not indicate relevant changes in the proportions of febrile illness on deployment, with trends pointing in different directions for soldiers and police officers. Interestingly, there was a tendency for a decrease in fever on deployment in the assessed German soldiers in contrast to an also non-significant increase in the assessed deployed German police officers during the pandemic period. It might be speculated that strict enforcement of infection control protocols within military field camps could have ensured a protection level against febrile illness, which could not be provided to police officers operating in dislocated settings apart from standardized field camp infrastructure. This assumption is in line with previous observations pointing towards more infections in deployed personnel operating without much infrastructural background [37].

Third, other than for febrile illness, there was a significance in deployed soldiers and a homologous trend in deployed police officers towards less gastroenteritis in the pandemic period. This is in striking contrast to the finding that there were no changed odds ratios for the diagnostic detection of microorganisms in post-deployment stool samples. This applied both for microbes with likely etiological relevance and for etiologically harmless protozoan colonizers, indicating the consumption of food and beverages under poor hygiene conditions. While this finding might be a mathematical phenomenon associated with the applied statistics for pathogens observed with low case numbers only, enteric protozoan parasites were so frequently recorded in stool samples of deployed soldiers that relevantly changed infection or colonization rates would most likely not have gone undetected. Therefore, we conclude that gastroenteric colonization or infection risks for deployed German soldiers and police officers did not relevantly change during the pandemic period in comparison to the pre-pandemic era.

Fourth, some pandemic-associated changes were recorded for indirect pathogen detection approaches, which were only available for post-deployment assessments of German soldiers. The recorded decline in *Mycobacterium tuberculosis*-specific immuno-conversion during military deployments in the pandemic period might well be explained by the pandemic-associated application of mechanical airway protection. The detection of less positive anti-malarial antibody titers during the pandemic period is less easy to explain. A generally increased awareness of infection risks alone seems to be a poor explanation, considering the lack of significance for declines in many other pathogen detections. In any case, even these two significant findings need to be interpreted with care. First, both diagnostic assessments of *M. tuberculosis*-specific immune-conversion and of anti-malarial antibodies to confirm previous malaria on deployment were not routine screening parameters, and so, their choice depended on individual medical decisions. Accordingly, varying judgments of presented medical conditions by different physicians are a likely source of bias. In addition, the recorded significances would not have passed the Bonferroni correction [38], an approach which was not applied in this explorative hypothesis-forming assessment.

As expected, because of the distribution of the deployment sites, most infections were associated with deployments to the WHO African region and the WHO Eastern Mediterranean region. Observed symptoms in patients colonized with apathogenic microorganisms matched the general distribution within the assessed populations and, insofar, did not point towards unexpected vulnerability. The recorded side findings of declined proportions of mefloquine-based antimalarial chemoprophylaxis with an associated increase in atovaquone–proguanil-based antimalarial chemoprophylaxis on military deployments reflected the impact of a German pharmaceutical authority warning (“Rote Hand Brief”) on neuropsychiatric side effects of mefloquine and the waving of timely limitations of prophylactic atovaquone-proguanil-use in the manufacturer’s recommendations within the study period [33]. Insofar, these findings were expected.

The study has a number of limitations. First, low overall infection rates limited the detectability of minor changes in infection risks for methodical reasons. Second, the screening approaches for soldiers and police officers were not identical for organizational reasons and so direct comparisons between these two populations were avoided. Third, considering the long total study period of more than 15 years, adaptations of diagnostic methods might have affected the comparability of individual test results, which is a common problem in retrospective long-term assessments. The same applies to adaptions of hygiene regimens for the deployment setting. Fourth, bias associated with individual diagnostic decisions by varying physicians over the assessment period cannot be denied. Fifth, the study design did not allow stratification by individual deployment-adapted infection control procedures during the COVID-19 pandemic but just provided a general overview. The same applies to the lack of information on individual adherence of the returnees with enforced infection control procedures, which is a likely source of bias, as suggested recently [26]. Sixth, no reliable deployment-associated denominators can be provided, because the count of deployed forces varied over time or was partly not available for confidentiality reasons. Accordingly, the calculations were just based on absolute numbers of medically assessed individuals. For the same reasons, no comparison with the local infectious disease situation at the deployment site is feasible. Seventh, the definition of the pandemic period focused on the years 2020–2022 might be an issue of debate. The choice was based on the period in which the strictest infection control procedures were enforced in Germany, which rapidly waned starting at the beginning of 2023 [32]. Eighth, no reliable data on deployment-related SARS-CoV-2 infections in the assessed soldiers and police officers were available as potential indicators of the general effectiveness of enforced infection control procedures on deployment. Ninth, varying proportions of soldiers and police officers returning from specific deployment sites in the pre-pandemic period and in the pandemic period are a likely source of bias.

## 5. Conclusions

In spite of the limitations stated above, the potential beneficial effects of COVID-19 pandemic-associated infection control procedures on the parameters included in the presented assessment have to be considered as low. The study contributes to scarcely available data on the medical effects of increased infection control procedures applied during the pandemic period in the deployment setting abroad. It can be considered as hypothesis-forming only and should be amended via further research including less holistic approaches. In particular, future interventional studies based on this experience should specifically address differences in the predominant mode of infectious disease transmission, e.g., differences between smear infections and predominantly food- or water-borne infections.

## Figures and Tables

**Table 1:** Comparison of the assessed deployed military populations in the pre-pandemic and pandemic periods.

	Pre-Pandemic (2006–2019)	Pandemic (2020–2022)	Difference in the Pre-Pandemic versus the Pandemic Situation
Rate of medically assessed military deployment events per year (n/year)	60.4/year (846/14 years)	54.7/year (164/3 years)	+5.7/year
Rate of post-deployment medically assessed soldiers per year (n/year)	55.1/year (772/14 years)	50.7/year (152/3 years)	+4.4/year
Female/male ratio (n:n)	1:14.8 (49:723)	1:15.9 (9:143)	n.a., odds ratio: 1.1
Mean age (years)	39.5	41.8	−2.3

n.a. = not applicable.

**Table 2:** Comparison of the assessed deployed police populations in the pre-pandemic and pandemic periods.

	Pre-Pandemic (2015–2019)	Pandemic (2020–2022)	Difference in the Pre-Pandemic versus the Pandemic Situation
Rate of medically assessed police deployment events per year, including pre- and post-deployment assessments (n/year)	23.2/year (116/5 years)	18.3/year (55/3 years)	+4.9/year
Rate of post-deployment medically assessed deployed police officers per year (n/year)	19.8/year (99/5 years)	11.7/year (35/3 years)	+8.1/year
Female/male ratio (n:n)	1:10.6 (10:106)	1:13.0 (4:52)	n.a., odds ratio: 1.2
Mean age (years)	42.8	46.9	−4.1

n.a. = not applicable.

**Table 3:** Deployment sites as reported by assessed military or police personnel in the pre-pandemic and the pandemic time period. Discrepancies in the total number of deployed individuals result from incomplete datasets or deployment to more than one site. If multiple countries or only not-further-specified geographic regions were reported as deployment sites, making any specific assignments of pathogen acquisition unfeasible, respective information was not provided in this table. Color code: No color = 0%, green = 0.1–5.0%, yellow = 5.1–20%, red = 20.1–100%.

Reported Deployment Sites	Pre-Pandemic (Military), n (%)	Pandemic (Military), n (%)	Pre-Pandemic (Police), n (%)	Pandemic (Police), n (%)
Afghanistan	41 (4.9%)	3 (2.1%)	4 (3.9%)	0 (0%)
Algeria	1 (0.1%)	0 (0%)	5 (4.9%)	4 (12.9%)
Argentina	2 (0.2%)	0 (0%)	0 (0%)	0 (0%)
Belize	1 (0.1%)	0 (0%)	0 (0%)	0 (0%)
Brazil	3 (0.4%)	0 (0%)	0 (0%)	0 (0%)
Burkina Faso	0 (0%)	3 (2.1%)	6 (5.8%)	2 (6.5%)
Burundi	0 (0%)	0 (0%)	1 (1.0%)	0 (0%)
Central African Republic	2 (0.2%)	0 (0%)	0 (0%)	0 (0%)
Chad	1 (0.1%)	1 (0.7%)	0 (0%)	0 (0%)
China	0 (0%)	0 (0%)	1 (1.0%)	1 (3.2%)
Costa Rica	0 (0%)	1 (0.7%)	0 (0%)	0 (0%)
Democratic Republic of the Congo	22 (2.6%)	0 (0%)	4 (3.9%)	1 (3.2%)
Djibouti	24 (2.9%)	4 (2.7%)	0 (0%)	0 (0%)
Egypt	0 (0%)	0 (0%)	6 (5.8%)	1 (3.2%)
Ethiopia	5 (0.6%)	1 (0.7%)	0 (0%)	0 (0%)
France	12 (1.4%)	0 (0%)	0 (0%)	0 (0%)
Gabon	6 (0.7%)	0 (0%)	0 (0%)	0 (0%)
Ghana	8 (1.0%)	5 (3.4%)	3 (2.9%)	0 (0%)
Guinea	1 (0.1%)	0 (0%)	0 (0%)	0 (0%)
Haiti	1 (0.1%)	0 (0%)	0 (0%)	0 (0%)
India	2 (0.2%)	0 (0%)	1 (1.0%)	0 (0%)
Indonesia	3 (0.4%)	0 (0%)	0 (0%)	0 (0%)
Iran	1 (0.1%)	0 (0%)	0 (0%)	0 (0%)
Iraq	19 (2.3%)	0 (0%)	3 (2.9%)	0 (0%)
Ivory Coast	0 (0%)	0 (0%)	2 (1.9%)	0 (0%)
Jordan	2 (0.2%)	1 (0.7%)	1 (1.0%)	0 (0%)
Kenya	4 (4.8%)	0 (0%)	5 (4.9%)	5 (16.1%)
Kosovo	1 (0.1%)	1 (0.7%)	0 (0%)	0 (0%)
Latvia	1 (0.1%)	2 (1.4%)	0 (0%)	0 (0%)
Lebanon	2 (0.2%)	1 (0.7%)	1 (1.0%)	0 (0%)
Liberia	4 (4.8%)	0 (0%)	0 (0%)	0 (0%)
Libya	0 (0%)	0 (0%)	1 (1.0%)	0 (0%)
Malaysia	0 (0%)	1 (0.7%)	0 (0%)	0 (0%)
Mali	211 (25.2%)	78 (53.4%)	10 (9.7%)	3 (9.7%)
Mauritania	1 (0.1%)	0 (0%)	5 (4.9%)	1 (3.2%)
Morocco	2 (0.2%)	0 (0%)	0 (0%)	0 (0%)
Niger	6 (0.7%)	9 (6.2%)	4 (3.9%)	0 (0%)
Nigeria	11 (1.3%)	4 (2.7%)	17 (16.5%)	7 (22.6%)
Pakistan	2 (0.2%)	1 (0.7%)	1 (1.0%)	0 (0%)
Peru	2 (0.2%)	0 (0%)	0 (0%)	0 (0%)
Russian Federation	0 (0%)	0 (0%)	1 (1.0%)	0 (0%)
Saudi Arabia	0 (0%)	0 (0%)	1 (1.0%)	0 (0%)
Senegal	9 (1.1%)	1 (0.7%)	6 (5.8%)	2 (6.5%)
Seychelles	1 (0.1%)	0 (0%)	0 (0%)	0 (0%)
Sierra Leone	0 (0%)	0 (0%)	1 (1.0%)	0 (0%)
Somalia	2 (0.2%)	0 (0%)	0 (0%)	0 (0%)
South Africa	0 (0%)	0 (0%)	3 (2.9%)	1 (3.2%)
Sri Lanka	0 (0%)	0 (0%)	1 (1.0%)	0 (0%)
Sudan (including South Sudan)	360 (43.0%)	23 (15.8%)	5 (4.9%)	1 (3.2%)
Sweden	0 (0%)	1 (0.7%)	0 (0%)	0 (0%)
Tanzania	4 (4.8%)	2 (1.4%)	0 (0%)	0 (0%)
Tunesia	0 (0%)	0 (0%)	4 (3.9%)	1 (3.2%)
Turkey	0 (0%)	2 (1.4%)	0 (0%)	1 (3.2%)
Uganda	55 (6.6%)	1 (0.7%)	0 (0%)	0 (0%)
Uzbekistan	2 (0.2%)	0 (0%)	0 (0%)	0 (0%)
Venezuela	1 (0.1%)	0 (0%)	0 (0%)	0 (0%)
Total	838 (100%)	146 (100%)	103 (100%)	31 (100%)

**Table 4:** Fever and gastroenteritis on military deployment. Significance was calculated by applying Fischer’s exact test. Significant differences are indicated in bold type.

Parameter	Pre-Pandemic Situation 2006–2019; n/n (%)	Pandemic Situation 2020–2022; n/n (%)	Odds Ratio (95%-CI)	Significance *p*
Fever	233/846 (27.5%)	36/164 (22.0%)	1.3 (0.9, 2.0)	0.1484
Gastroenteritis	519/846 (61.3%)	33/164 (20.1%)	**6.3 (4.2, 9.5)**	**<0.0001**

*p* was calculated using Fisher’s exact test (two-sided). Odds ratio-associated 95% confidence intervals (95%-CI) were calculated using the approximation of Woolf.

**Table 5:** Fever and gastroenteritis on police deployment (pre-deployment assessments not shown in the table). Significance was calculated by applying Fischer’s exact test.

Parameter	Pre-Pandemic Situation 2006–2019; n/n (%)	Pandemic Situation 2020–2022; n/n (%)	Odds Ratio (95%-CI)	Significance *p*
Fever	8/99 (8.1%)	6/35 (17.1%)	0.4 (0.1, 1.3)	0.1945
Gastroenteritis	35/99 (35.4%)	9/35 (25.7%)	1.5 (0.7, 3.7)	0.4026

*p* was calculated using Fisher’s exact test (two-sided). Odds ratio-associated 95% confidence intervals (95%-CI) were calculated using the approximation of Woolf.

**Table 6:** Direct and indirect detection of pathogens in deployed soldiers. Only parameters for which at least one positive signal was detected are shown. Significance was calculated by applying Fischer’s exact test. Significant results are indicated in bold type.

Parameter	Pre-Pandemic Situation 2006–2019; n/n (%)	Pandemic Situation 2020–2022; n/n (%)	Odds Ratio (95%-CI)	Significance *p*
Direct proof of microorganisms (PCR, microscopy or culture)				
*Blastocystis hominis*	204/846 (24.1%)	38/164 (23.2%)	1.1 (0.7, 1.6)	0.8420
*Entamoeba* spp. (without *Entamoeba histolytica*)	63/846 (7.4%)	6/164 (3.7%)	2.1 (0.9, 5.0)	0.0903
*Giardia duodenalis*	23/846 (2.7%)	5/164 (3.0%)	0.9 (0.3, 2.4)	0.7951
*Endolimax nana*	13/846 (1.5%)	5/164 (3.0%)	0.5 (0.2, 1.4)	0.1934
*Dientamoeba fragilis*	7/846 (0.8%)	4/164 (2.4%)	0.3 (0.1, 1.2)	0.0873
*Plasmodium* spp.	7/846 (0.8%)	0/164 (0%)	3.0 (0.2, 51.8)	0.6062
*Yersinia enterocolitica*	2/846 (0.2%)	0/164 (0%)	1.0 (<0.1, 20.4)	1.0000
*Campylobacter jejuni*	1/846 (0.1%)	0/164 (0%)	0.6 (<0.1, 20.4)	1.0000
*Schistosoma* spp.	1/846 (0.1%)	0/164 (0%)	0.6 (<0.1, 20.4)	1.0000
*Strongyloides stercoralis*	2/846 (0.2%)	1/164 (0.6%)	0.4 (<0.1, 4.3)	0.4127
*Leishmania* spp. (in a skin smear)	1/846 (0.1%)	0/164 (0%)	0.6 (<0.1, 20.4)	1.0000
Indirect proof of microorganisms (serology or interferon-gamma release assay (IGRA))				
Anti-*Plasmodium* spp.-antibodies	35/846 (4.1%)	0/164 (0%)	**14.4 (0.9, 236.0)**	**0.0037**
Anti-*Schistosoma* spp. antibodies	15/846 (1.8%)	5/164 (3.0%)	0.6 (0.2, 1.6)	0.3514
Anti-*Yersinia enterocolitica* antibodies	3/846 (0.4%)	0/164 (0%)	1.4 (<0.1, 26.6)	1.0000
Anti-*Strongyloides stercoralis* antibodies	2/846 (0.2%)	1/164 (0.6%)	1.0 (<0.1, 20.4)	1.0000
Anti-*Leishmania* spp. antibodies	1/846 (0.1%)	0/164 (0%)	0.6 (<0.1, 20.4)	1.0000
*Mycobacterium tuberculosis*-specific IGRA	29/846 (3.4%)	0/164 (0%)	**11.9 (0.7, 195.4)**	**0.0090**
*Mycobacterium tuberculosis*-specific IGRA resulting in isoniazide-prophylaxis	8/846 (0.9%)	0/164 (0%)	3.3 (0.2, 58.1)	0.3671

*p* was calculated using Fisher’s exact test (two-sided). Odds ratio-associated 95% confidence intervals (95%-CI) were calculated using the approximation of Woolf.

**Table 7:** Direct detection of pathogens in deployed police officers (pre-deployment assessments not shown in the table). Only parameters for which at least one positive signal was detected are shown.

Parameter	Pre-Pandemic Situation 2006–2019; n/n (%)	Pandemic Situation 2020–2022; n/n (%)	Odds Ratio (95%-CI)	Significance *p*
Direct proof of microorganisms (PCR, microscopy or culture)				
*Giardia duodenalis*	3/99 (3.0%)	0/35 (0%)	2.6 (0.1, 51.1)	0.5670
*Campylobacter jejuni*	2/99 (2.0%)	0/35 (0%)	1.8 (<0.1, 38.9)	1.0000
*Salmonella* spp.	1/99 (1.0%)	0/35 (0%)	1.1 (<0.1, 27.2)	1.0000
*Shigella* spp./enteroinvasive *Escherichia coli*	3/99 (3.0%)	0/35 (0%)	2.6 (0.1, 51.1)	0.5670
*Strongyloides stercoralis*	2/99 (2.0%)	0/35 (0%)	1.8 (<0.1, 38.9)	1.0000
*Blastocystis hominis* ^#^	0/99 (0%)	1/35 (2.9%)	0.1 (<0.1, 2.9)	0.2612
*Vibrio* spp. ^#^	0/99 (0%)	1/35 (2.9%)	0.1 (<0.1, 2.9)	0.2612

*p* was calculated using Fisher’s exact test (two-sided). Odds ratio-associated 95% confidence intervals (95%-CI) were calculated using the approximation of Woolf. ^#^ Parameter not included in the assessment earlier than in 2022.

**Table 8:** Choice of the antimalarial drug or drug combination in soldiers reporting antimalarial chemoprophylaxis. Significance was calculated by applying Fischer’s exact test.

Parameter	Pre-Pandemic Situation 2006–2019; n/n (%)	Pandemic Situation 2020–2022; n/n (%)	Odds Ratio (95%-CI)	Significance *p*
Mefloquine	334/614 (54.4%)	0/25 (0%)	**60.8 (3.7, 1004.2)**	**<0.0001**
Atovaquone/proguanil	252/614 (41.0%)	25/25 (100%)	**<0.1 (<0.1, 0.2)**	**<0.0001**
Doxycycline	28/614 (4.6%)	0/25 (0%)	2.5 (0.1, 41.8)	0.6195

*p* was calculated using Fisher’s exact test (two-sided). Odds ratio-associated 95% confidence intervals (95%-CI) were calculated using the approximation of Woolf. Significant differences are indicated in bold type.

## Data Availability

All relevant data have been provided in the manuscript. Raw data can be made available upon reasonable request.

## Appendix A

**Table A1:** Associations of direct or indirect pathogen detection in returned soldiers and the WHO regions of the deployment sites. “Uncertain region of infection” defines situations in which a likely assignment was unfeasible due to deployments to various geographic regions.

Microorganism	African Region	Region of the Americas	South East Asia Region	European Region	Eastern Mediterranean Region	Uncertain Region of Infection	Total
*Blastocystis hominis*	104 (43.0%)	2 (0.8%)	3 (1.2%)	6 (2.5%)	127 (52.5%)	0 (0%)	242 (100%)
*Entamoeba* spp. (without *Entamoeba histolytica*)	20 (29.0%)	0 (0%)	0 (0%)	0 (0%)	47 (68.1%)	2 (2.9%)	69 (100%)
*Giardia duodenalis*	8 (28.6%)	0 (0%)	0 (0%)	0 (0%)	19 (67.9%)	1 (3.6%)	28 (100%)
*Endolimax nana*	8 (44.4%)	0 (0%)	1 (5.6%)	0 (0%)	9 (50%)	0 (0%)	18 (100%)
*Dientamoeba fragilis*	4 (36.4%)	0 (0%)	0 (0%)	0 (0%)	7 (63.6%)	0 (0%)	11 (100%)
*Plasmodium* spp. (direct and indirect proof)	12 (32.4%)	0 (0%)	0 (0%)	0 (0%)	25 (67.6%)	0 (0%)	37 (100%)
*Yersinia enterocolitica* (direct and indirect proof)	0 (0%)	0 (0%)	0 (0%)	0 (0%)	0 (0%)	5 (100%)	5 (100%)
*Campylobacter jejuni*	1 (100%)	0 (0%)	0 (0%)	0 (0%)	0 (0%)	0 (0%)	1 (100%)
*Schistosoma* spp. (direct and indirect proof)	4 (20%)	0 (0%)	2 (10%)	0 (0%)	12 (60%)	2 (10%)	20 (100%)
*Strongyloides stercoralis* (direct & indirect proof)	4 (66.7%)	0 (0%)	1 (16.7%)	0 (0%)	1 (16.7%)	0 (0%)	6 (100%)
*Leishmania* spp. (skin smear and serology)	0 (0%)	0 (0%)	0 (0%)	0 (0%)	0 (0%)	1 (100%)	1 (100%)
*Mycobacterium tuberculosis*-specific IGRA resulting in isoniazide-prophylaxis *	2 (25%)	0 (0%)	0 (0%)	0 (0%)	6 (75%)	0 (0%)	8 (100%)
*Salmonella* spp.	0 (0%)	0 (0%)	0 (0%)	0 (0%)	0 (0%)	1 (100%)	1 (100%)

* Non-prophylaxis-associated results were not considered because temporal association to the deployment scenario was considered uncertain.

**Table A2:** Associations of direct pathogen detection in returned police officers and the WHO regions of the deployment sites.

Microorganism	African Region	Region of the Americas	South East Asia Region	European Region	Eastern Mediterranean Region	Total
*Giardia duodenalis*	3 (100%)	0 (0%)	0 (0%)	0 (0%)	0 (0%)	3 (100%)
*Campylobacter jejuni*	0 (0%)	0 (0%)	0 (0%)	0 (0%)	2 (100%)	2 (100%)
*Salmonella* spp.	0 (0%)	0 (0%)	0 (0%)	0 (0%)	1 (100%)	1 (100%)
*Shigella* spp./enteroinvasive *Escherichia coli*	3 (100%)	0 (0%)	0 (0%)	0 (0%)	0 (0%)	1 (100%)
*Strongyloides stercoralis*	1 (50%)	0 (0%)	0 (0%)	0 (0%)	1(50%)	2 (100%)
*Blastocystis hominis*	1 (100%)	0 (0%)	0 (0%)	0 (0%)	0 (0%)	1 (100%)
*Vibrio* spp.	1 (100%)	0 (0%)	0 (0%)	0 (0%)	0 (0%)	1 (100%)

**Table A3:** Associations of direct or indirect pathogen detection of the deployed soldiers and recorded symptoms.

Direct or Indirect Pathogen Detection	Fever	Gastroenteritis	Total (per Cent)
*Blastocystis hominis*	59 (24.4%)	143 (59.1%)	242 (100%)
*Entamoeba* spp. (without *Entamoeba histolytica*)	26 (37.7%)	42 (60.9%)	69 (100%)
*Giardia duodenalis*	7 (25%)	25 (89.3%)	28 (100%)
*Endolimax nana*	7 (38.9%)	11 (61.1%)	18 (100%)
*Dientamoeba fragilis*	5 (45.5%)	5 (45.5%)	11 (100%)
*Plasmodium* spp. (direct and indirect proof)	5 (13.5%)	1 (2.7%)	37 (100%)
*Yersinia enterocolitica* (direct and indirect proof)	3 (60%)	5 (100%)	5 (100%)
*Campylobacter jejuni*	0 (0%)	1 (100%)	1 (100%)
*Schistosoma* spp. (direct and indirect proof)	5 (25%)	8 (40%)	20 (100%)
*Strongyloides stercoralis* (direct and indirect proof)	3 (50%)	4 (66.7%)	6 (100%)
*Leishmania* spp. (skin smear and serology)	0 (0%)	1 (100%)	1 (100%)
*Mycobacterium tuberculosis*-specific IGRA resulting in isoniazide-prophylaxis *	5 (62.5%)	6 (75%)	8 (100%)
*Salmonella* spp.	1 (100%)	1 (100%)	1 (100%)

* Non-prophylaxis-associated results were not considered because temporal association to the deployment scenario was considered uncertain.

**Table A4:** Associations of direct pathogen detection police officers returned from deployment and recorded symptoms.

Direct or Indirect Pathogen Detection	Fever	Gastroenteritis	Total
*Giardia duodenalis*	1 (33.3%)	2 (66.7%)	3 (100%)
*Campylobacter jejuni*	0 (0%)	1 (50%)	2 (100%)
*Salmonella* spp.	0 (0%)	0 (0%)	1 (100%)
*Shigella* spp./enteroinvasive *Escherichia coli*	0 (0%)	2 (66.7%)	1 (100%)
*Strongyloides stercoralis*	0 (0%)	1 (50%)	2 (100%)
*Blastocystis hominis*	0 (0%)	0 (0%)	1 (100%)
*Vibrio* spp.	0 (0%)	0 (0%)	1 (100%)
